# Supplementary material for: Values Clarification as a Reflective Practice for Preclerkship Medical Students
Source: MedEdPORTAL. 2023 May 2;19:11308. doi: 10.15766/mep_2374-8265.11308 (PMC10151448; doi:10.15766/mep_2374-8265.11308)
Supplement: Supplementary file 1 — Workshop Syllabus.docxExercise.docxWorkshop Introduction.pptxWorkshop Implementation Guide.docxPostsession Survey.docx [file mep_2374-8265.11308-s001.zip › A. Workshop Syllabus.docx]

**Appendix A.** Values Clarification Workshop: Syllabus

**Required Preparation**

1. Complete the values clarification exercise. When you are done, **be sure to think about what you would like to share in your small group**.
2. Stahl RY & Emanuel EJ, Physicians, Not Conscripts — Conscientious Objection in Health Care, *N Engl J Med* (2017) 376;14, 1380-1385

**Goal**

To alert students to the possibility that the professional expectations placed on them may elicit significant moral stress and to demonstrate to them that values clarification can help them engage with and work through the moral discomfort with professional responsibilities they may experience.

**Learning Objectives**

By the end of this workshop, learners will be able to:

1. Describe the origin and nature of their beliefs about two or more of the following topics: pregnancy termination, male circumcision, continuation/withdrawal of life-sustaining treatment, hormone therapy for transgender youth, physician aid in dying, and involuntary hospitalization
2. Outline general steps to take when navigating a situation in which their professional responsibilities are in conflict with their personal moral beliefs and explain specifically how values clarification fits into this approach
3. Describe the ways in which their beliefs align or differ from those held by members of their discussion group regarding one or more of the six healthcare topics listed above.

**Session Details**

**Large Group:**

10 minutes

Presentation: Introduction to values clarification

35 minutes

Physician panel

**Small Group:**

5 minutes

Safe space guidelines: Given the sensitive nature of some of the topics addressed in this session, the facilitator or a student volunteer should ask group members to:

- give all members the space to share their reactions and thoughts
- use appropriate and respectful language at all times
- respect confidentiality (If someone shares a story that they would like to keep confidential, it stays within and doesn’t leave the small group.)
- refrain from pressing anyone to share personal experiences that may make them feel uncomfortable

10 minutes

Check in. Group members are invited to say a few words about how they are doing and what they thought about the large group session. In addition, they should share which topics they explored in the values clarification exercise and which topics they are most interested in discussing with the group.

40 minutes

Groups should choose two or three topics to discuss, with at least one coming from the following list:

- Pregnancy termination
- Continuation/withdrawal of life-sustaining treatment
- Hormone therapy for transgender youth
- Physician aid in dying
- Involuntary hospitalization
- Male circumcision

For each topic being discussed, consider the following prompts:

- What, if anything, did you learn about your beliefs about this topic from doing the values clarification exercise?
- Did completing the exercise have an impact on your comfort with this topic, and if so, in what way?
- Have you experienced, or do you anticipate experiencing, any moral discomfort around the professional expectations associated with this topic? What is the basis of the discomfort?
- What approaches did you, or might you, take to address this discomfort? What is the role of values clarification in the process of resolving this discomfort? .

The small group discussion is intended to give members of the group a chance to share their perspectives and concerns about healthcare topics that they may find ethically challenging, to learn about the points of view and experiences of others in the group, to further clarify the nature of any conflict with professional expectations they have experienced or are envisioning, and to consider what steps they might take to manage a professional situation that pushes up against their personal values. The goal is for this sharing and exploration to take place in a safe environment. **Please note: The discussion should not, under any circumstances, turn into a debate about the ethical merits of one or another point of view.**

10 minutes

Check out with take-away points
