# Supplementary material for: Values Clarification as a Reflective Practice for Preclerkship Medical Students
Source: MedEdPORTAL. 2023 May 2;19:11308. doi: 10.15766/mep_2374-8265.11308 (PMC10151448; doi:10.15766/mep_2374-8265.11308)
Supplement: Supplementary file 1 — Workshop Syllabus.docxExercise.docxWorkshop Introduction.pptxWorkshop Implementation Guide.docxPostsession Survey.docx [file mep_2374-8265.11308-s001.zip › B. Exercise.docx]

**Appendix B.** Values Clarification Exercise

We use the term “values” to signify the collection of deeply-held moral beliefs and priorities that each of us holds. In other words, our values are the beliefs that we have about what is important in life and that inform us of what the right thing to do is in any given situation. Sometimes, the choices we face challenge these beliefs. In such a circumstance, gaining an understanding of the origin and nature of our values is an important first step towards

1. identifying what exactly is the basis of the discomfort or conflict we are experiencing and
2. making decisions about what compromises are or are not acceptable and what steps more generally to take to resolve the situation.

This exercise is offered as an example of how you might go about exploring and clarifying your own values.

Note that the discussion that you will have with your small group during the workshop will focus on the experience of completing this exercise and on experiences, or anticipated experiences, of discomfort with professional expectations. Please prepare accordingly.

There are many topics in healthcare that people have strong beliefs about. Examples include

- Pregnancy termination
- Continuation/withdrawal of life-sustaining treatment
- Hormone therapy for transgender youth
- Physician aid in dying
- Involuntary hospitalization
- Male circumcision

Please reflect on the following questions. Space is provided for written notes. The expected time to complete this exercise is approximately 60 minutes.

**External Influences**

- What social groups and social identities have influenced your personal values? In what ways do your current values align with those of the social groups and identities that have influenced you and in what ways do they differ?
- How frequently do you consciously refer to your spiritual beliefs before making a decision? Have you held the same spiritual beliefs since childhood? Have challenging life circumstances ever made you act against your spiritual beliefs, or alter them?
- If you were to have a discussion with yourself five years ago, what topics/issues would you disagree on? Are any of these topics/issues related to the healthcare topics listed above?
- How have the influences that you just identified in your life affected your beliefs about the healthcare topics listed above? Be sure to consider at least two of these topics in depth.

### **Personal Experiences**

- Which of the healthcare topics listed above has had a significant impact on you, either because of direct experience or that of someone close to you? What is that impact?
- If you have had exposure to any of these topics:
  - What were the most difficult aspects of any decisions that you were involved in making?
  - What, if anything, would you change about your experiences?
- Imagine working with a patient who is in a situation related to one of the topics you have had experience with. Do you think your experience would complicate or support your ability to understand your patient’s experience and choices? In what ways?

### **Professional Obligations and Role of Healthcare Provider**

Evaluating our professional obligations in certain situations can be a challenge. It can help if we take the time to ask ourselves some questions about the relationship between our personal views and our professional role.

Choose three topics that elicit some discomfort when you consider providing care related to the topic. They do not have to be from the list at the top of this exercise. For each topic, consider the sets of questions that follow. Though there may be overlap with earlier questions, note that the focus here is on the discomfort you feel about a certain clinical scenario.

1. What are your beliefs and values about this topic? How do the influences you considered earlier and the experiences you have had with this topic contribute to the formation of these beliefs and values? To what extent are you unsure about your beliefs and values?
2. What are the generally accepted professional obligations for a physician in the scenario you are considering?
3. To what extent is the discomfort you feel due to tension between your personal beliefs and values and your desire to fulfill your professional responsibilities?
   - 1. If such tension is present, how would you try to reduce it?
     2. Where would you turn for support?
     3. What would be the “ideal” way to deal with the situation?

When you have completed the exercise, take a few minutes to write down what, if anything, surprised you while working through this values clarification worksheet. Would it be meaningful to you and of benefit to your small group for you to share these surprises with the group?
