# Supplementary material for: Values Clarification as a Reflective Practice for Preclerkship Medical Students
Source: MedEdPORTAL. 2023 May 2;19:11308. doi: 10.15766/mep_2374-8265.11308 (PMC10151448; doi:10.15766/mep_2374-8265.11308)
Supplement: Supplementary file 1 — Workshop Syllabus.docxExercise.docxWorkshop Introduction.pptxWorkshop Implementation Guide.docxPostsession Survey.docx [file mep_2374-8265.11308-s001.zip › C. Workshop Introduction.pptx]

## Slide 1
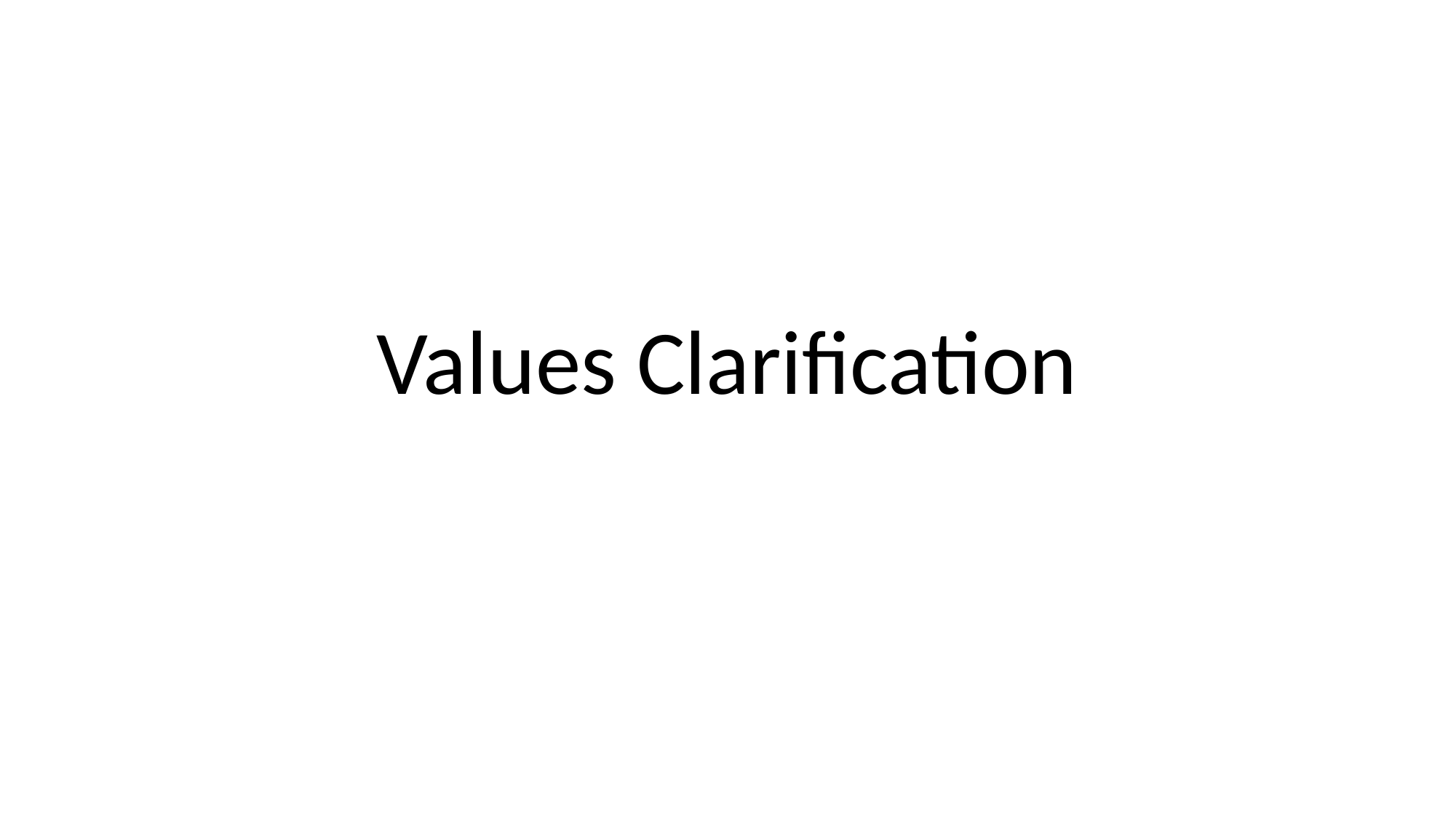

# Values Clarification

## Slide 2
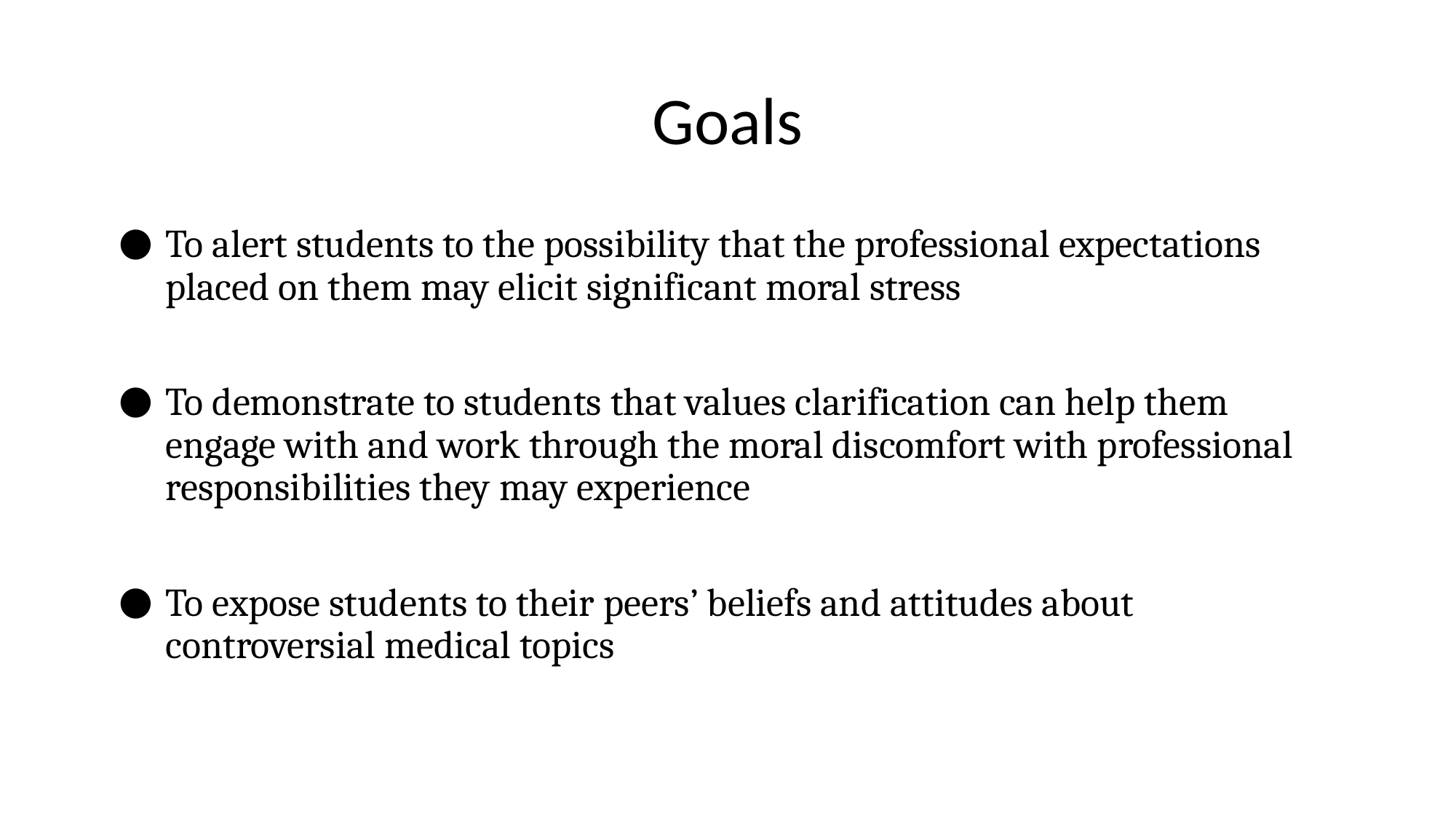

# Goals
To alert students to the possibility that the professional expectations placed on them may elicit significant moral stress
To demonstrate to students that values clarification can help them engage with and work through the moral discomfort with professional responsibilities they may experience
To expose students to their peers’ beliefs and attitudes about controversial medical topics

## Slide 3
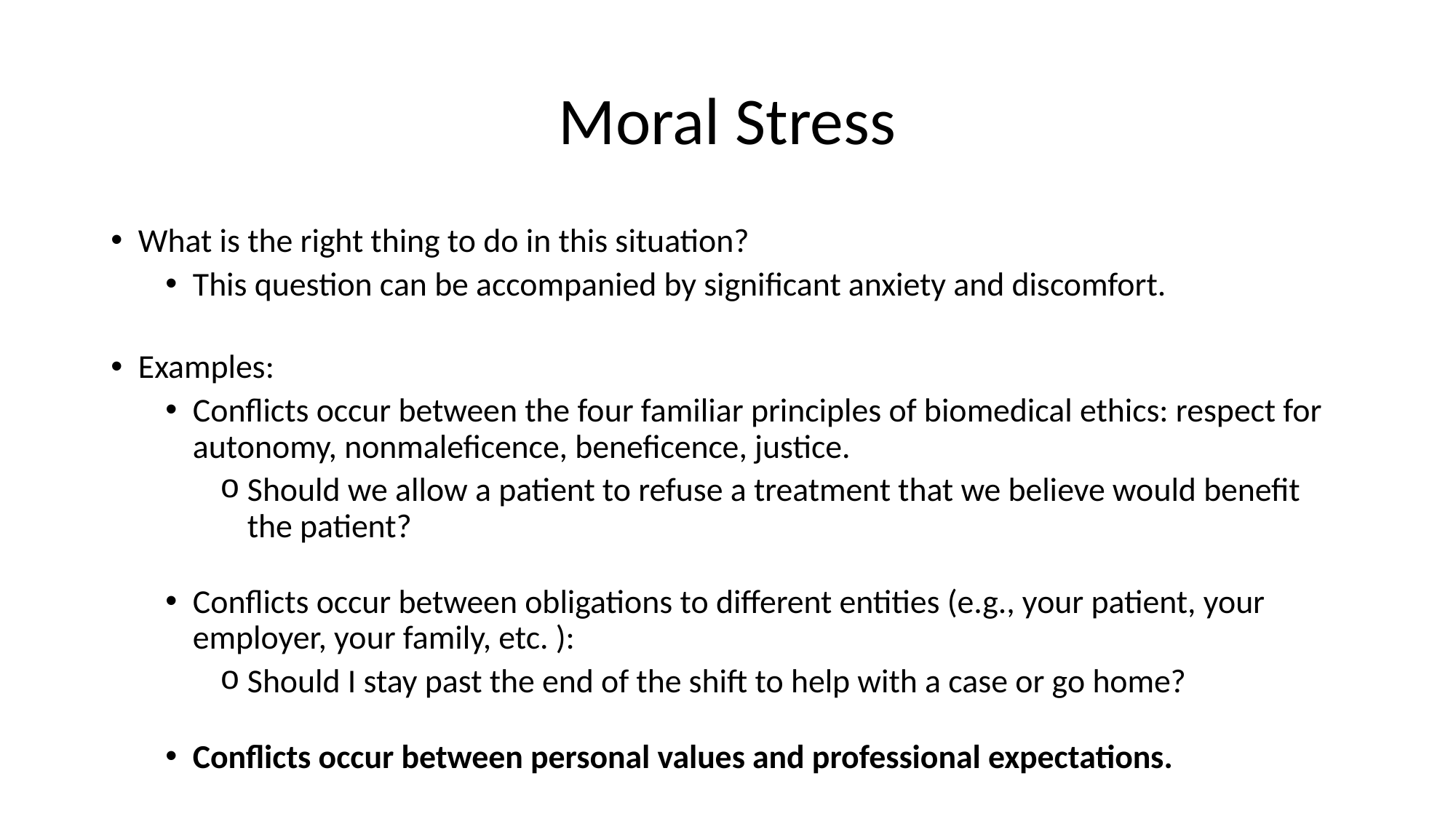

# Moral Stress
What is the right thing to do in this situation?
This question can be accompanied by significant anxiety and discomfort.
Examples:
Conflicts occur between the four familiar principles of biomedical ethics: respect for autonomy, nonmaleficence, beneficence, justice.
Should we allow a patient to refuse a treatment that we believe would benefit the patient?
Conflicts occur between obligations to different entities (e.g., your patient, your employer, your family, etc. ):
Should I stay past the end of the shift to help with a case or go home?
Conflicts occur between personal values and professional expectations.

## Slide 4
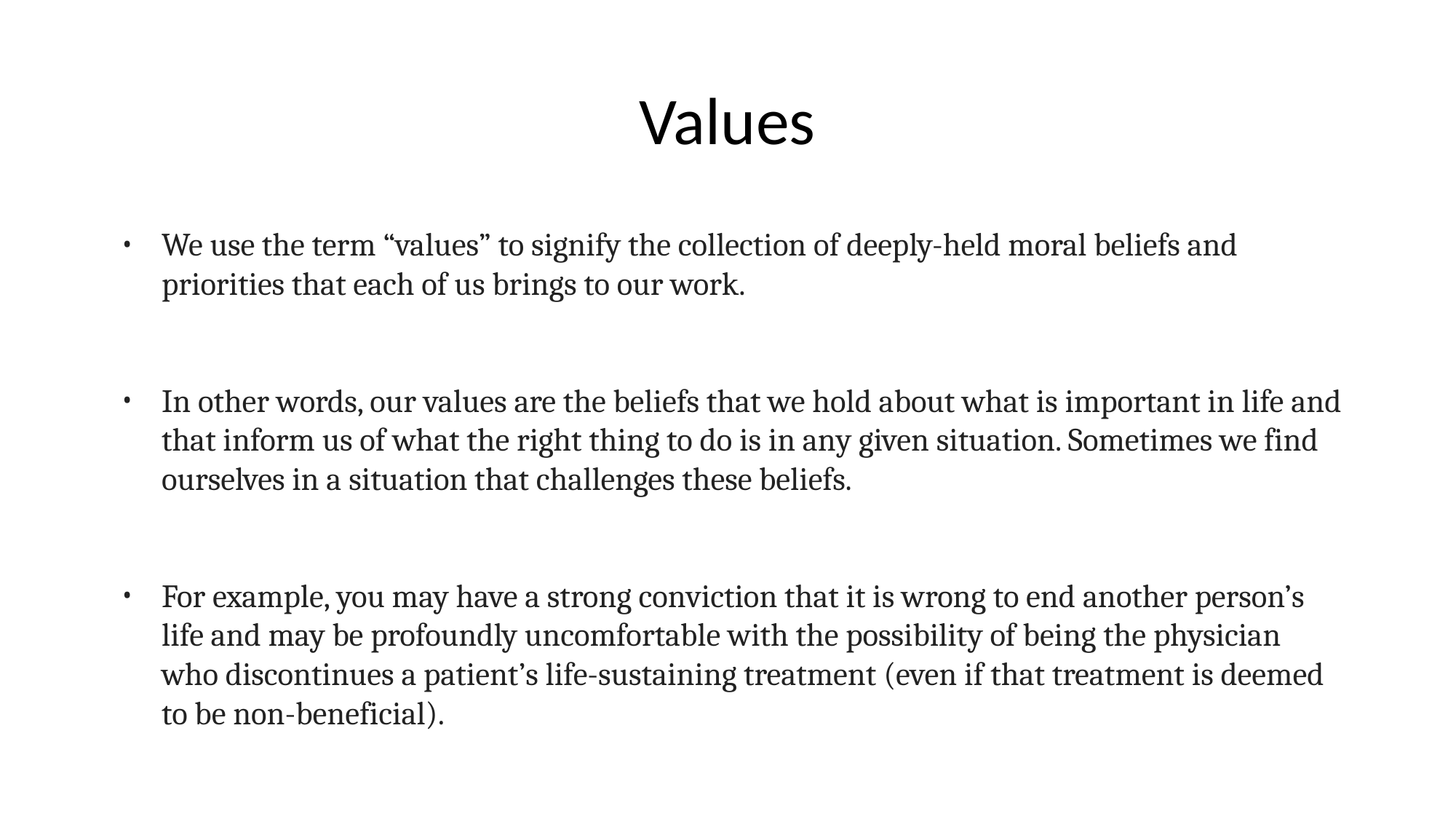

# Values
We use the term “values” to signify the collection of deeply-held moral beliefs and priorities that each of us brings to our work.
In other words, our values are the beliefs that we hold about what is important in life and that inform us of what the right thing to do is in any given situation. Sometimes we find ourselves in a situation that challenges these beliefs.
For example, you may have a strong conviction that it is wrong to end another person’s life and may be profoundly uncomfortable with the possibility of being the physician who discontinues a patient’s life-sustaining treatment (even if that treatment is deemed to be non-beneficial).

## Slide 5
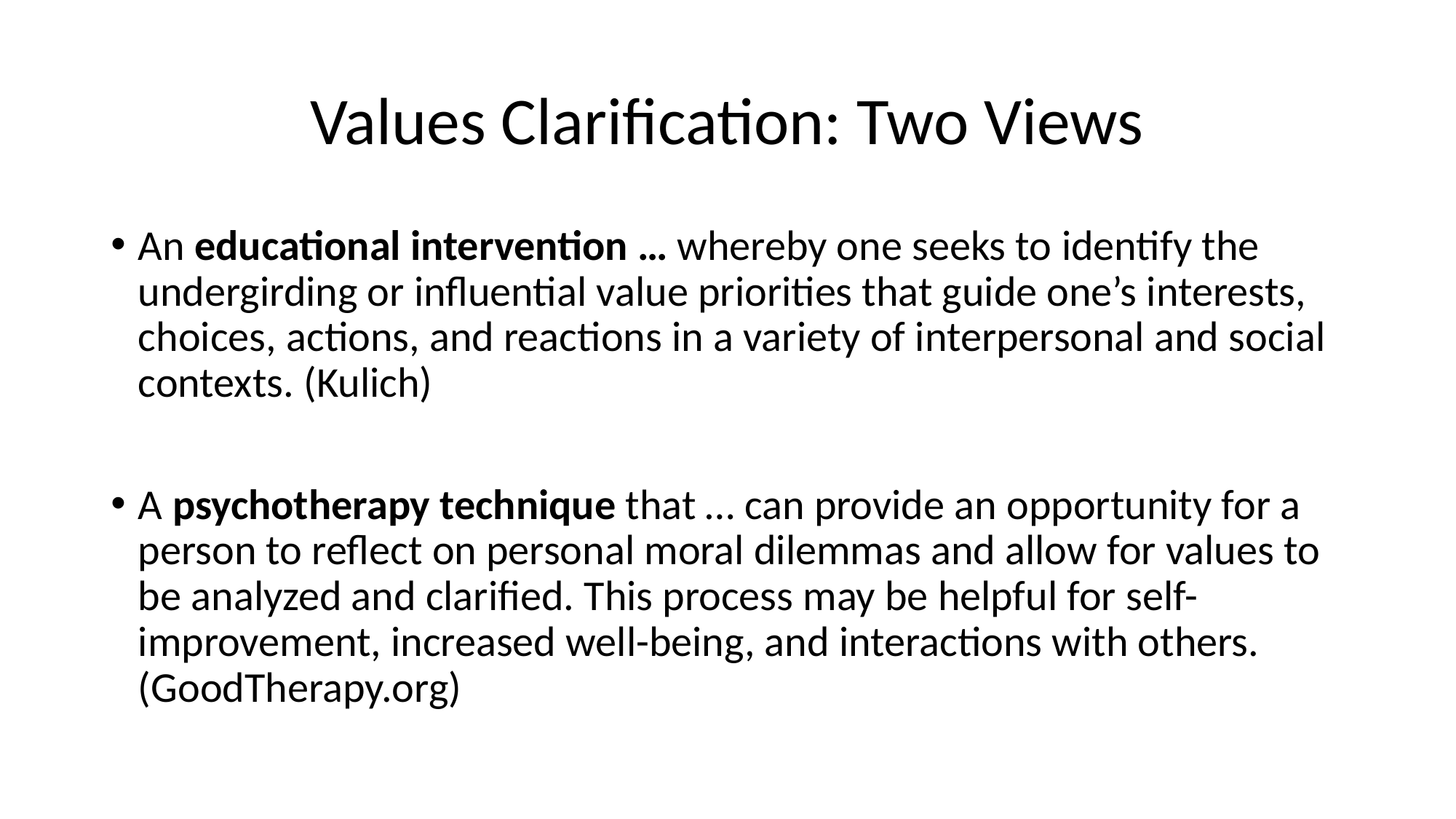

# Values Clarification: Two Views
An educational intervention … whereby one seeks to identify the undergirding or influential value priorities that guide one’s interests, choices, actions, and reactions in a variety of interpersonal and social contexts. (Kulich)
A psychotherapy technique that … can provide an opportunity for a person to reflect on personal moral dilemmas and allow for values to be analyzed and clarified. This process may be helpful for self-improvement, increased well-being, and interactions with others. (GoodTherapy.org)

## Slide 6
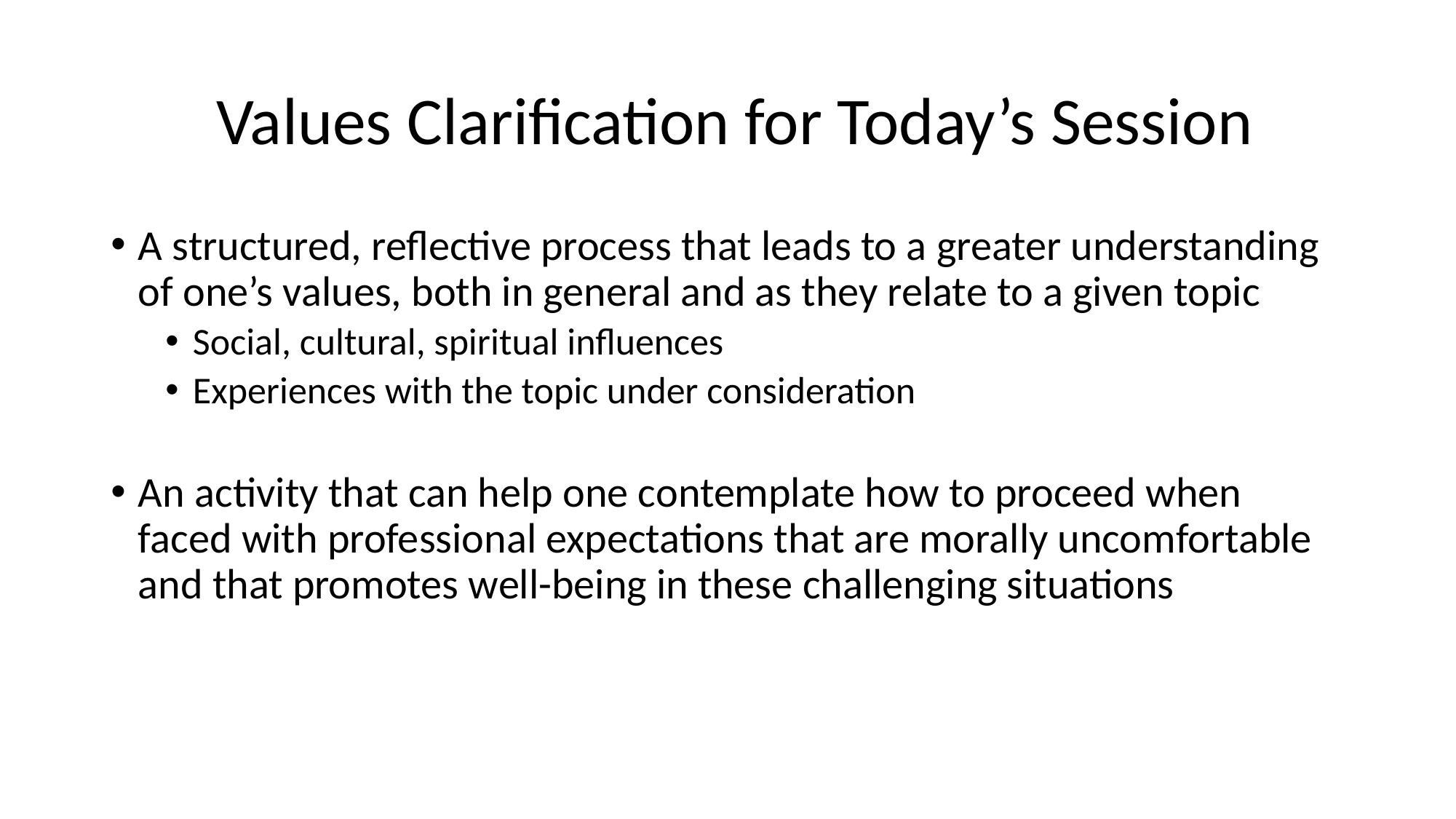

# Values Clarification for Today’s Session
A structured, reflective process that leads to a greater understanding of one’s values, both in general and as they relate to a given topic
Social, cultural, spiritual influences
Experiences with the topic under consideration
An activity that can help one contemplate how to proceed when faced with professional expectations that are morally uncomfortable and that promotes well-being in these challenging situations

## Slide 7
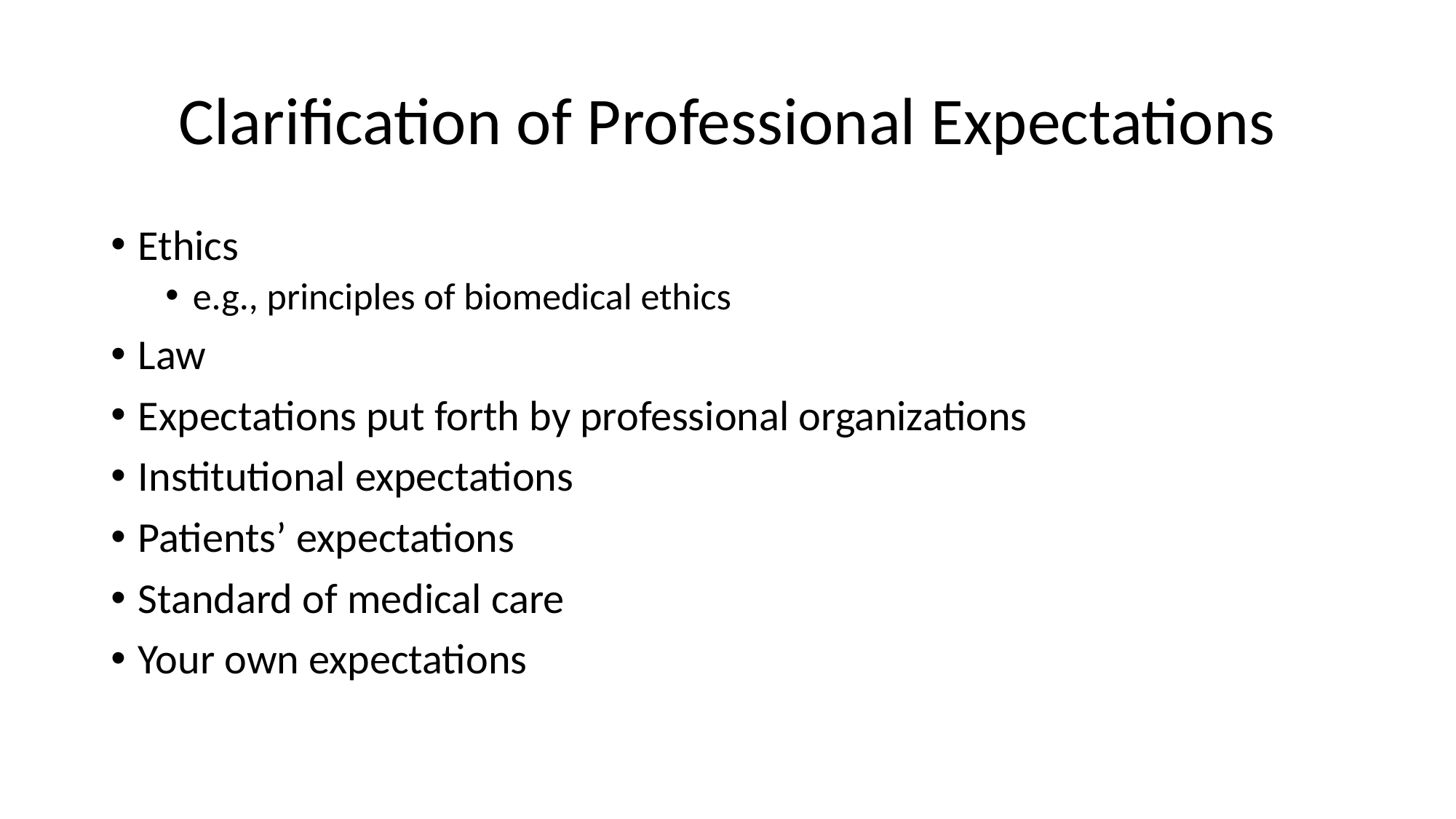

# Clarification of Professional Expectations
Ethics
e.g., principles of biomedical ethics
Law
Expectations put forth by professional organizations
Institutional expectations
Patients’ expectations
Standard of medical care
Your own expectations

## Slide 8
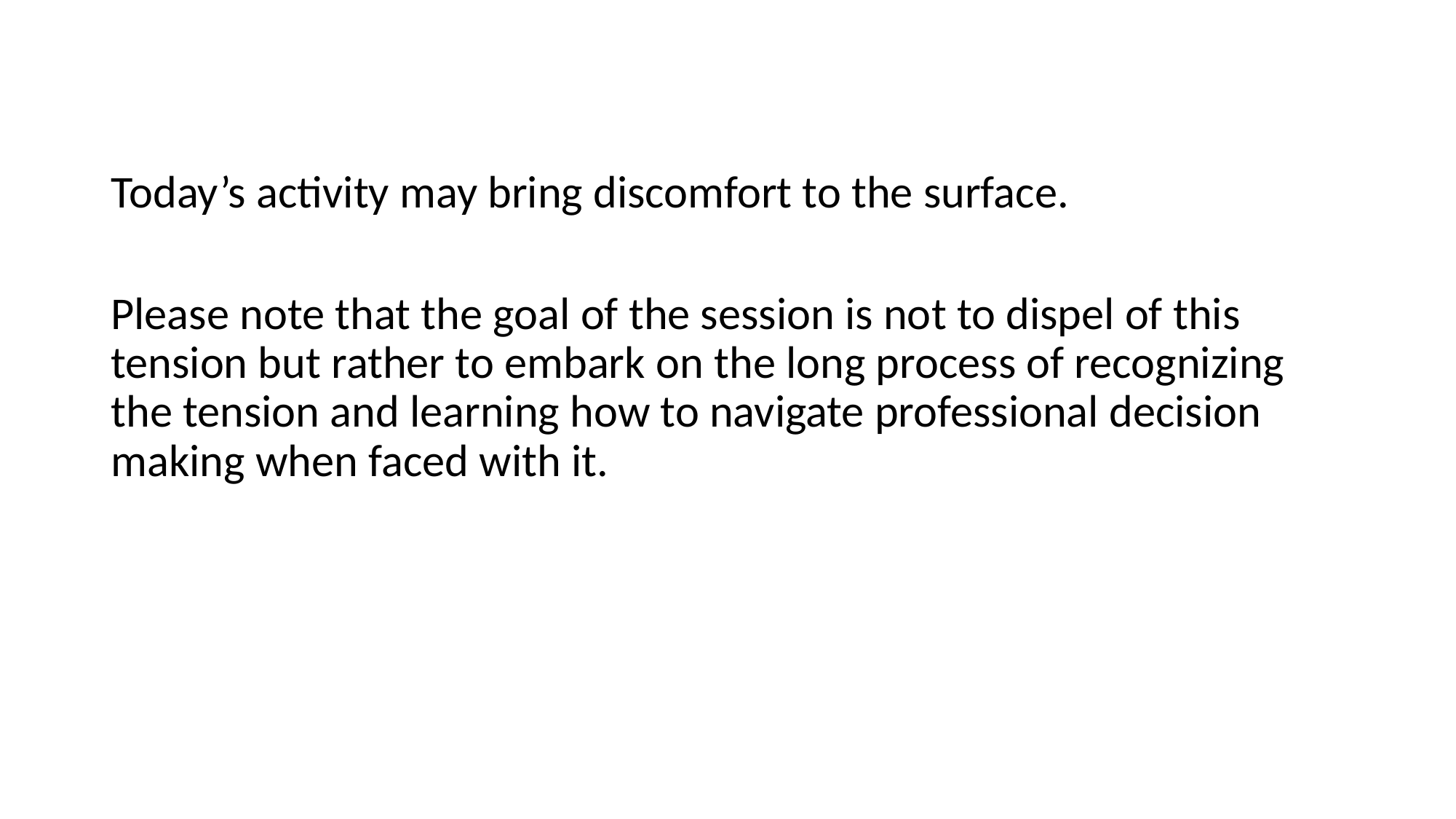

Today’s activity may bring discomfort to the surface.
Please note that the goal of the session is not to dispel of this tension but rather to embark on the long process of recognizing the tension and learning how to navigate professional decision making when faced with it.

## Slide 9
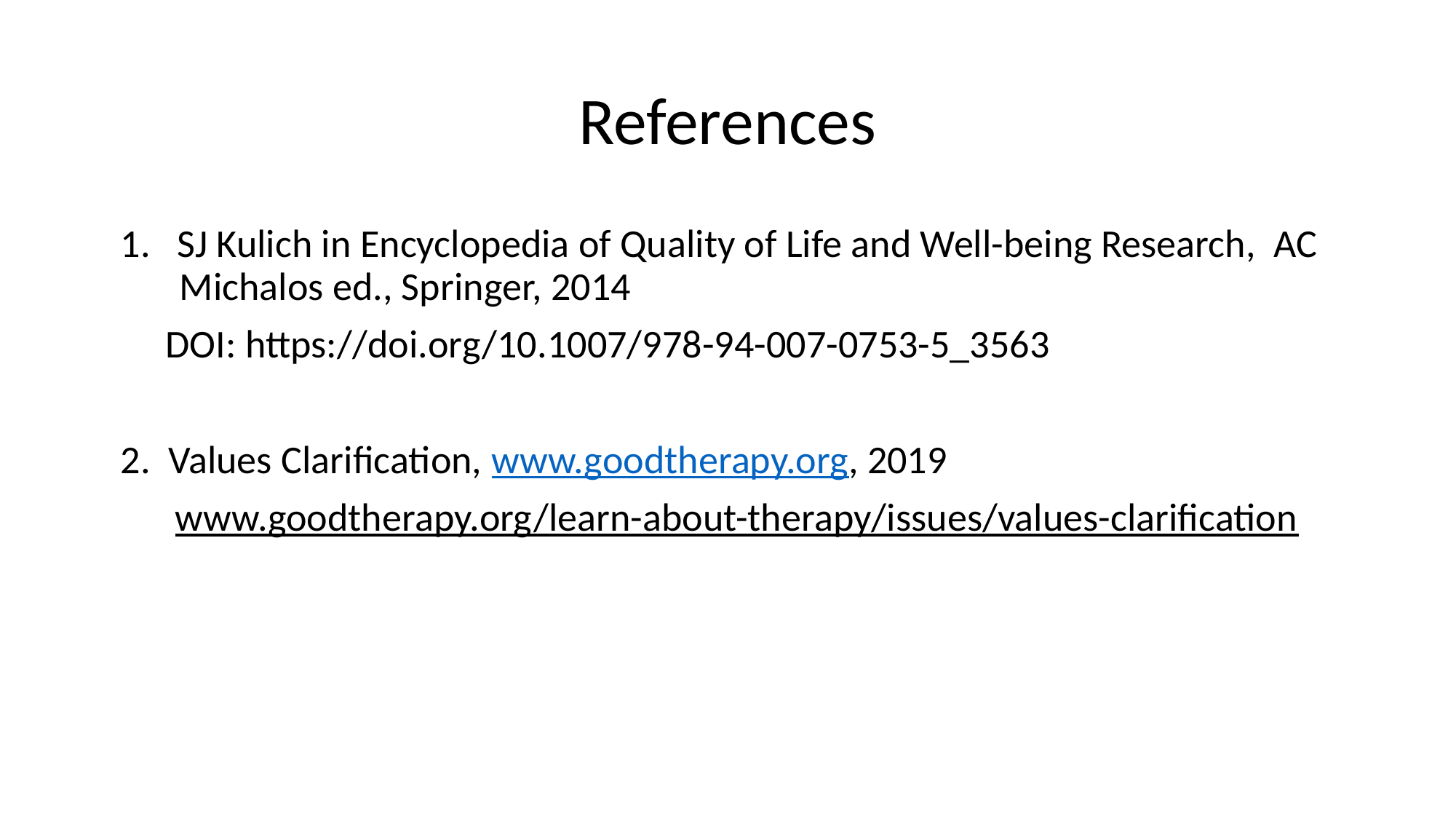

# References
1. SJ Kulich in Encyclopedia of Quality of Life and Well-being Research, AC Michalos ed., Springer, 2014
 DOI: https://doi.org/10.1007/978-94-007-0753-5_3563
2. Values Clarification, www.goodtherapy.org, 2019
www.goodtherapy.org/learn-about-therapy/issues/values-clarification
