# Supplementary material for: Values Clarification as a Reflective Practice for Preclerkship Medical Students
Source: MedEdPORTAL. 2023 May 2;19:11308. doi: 10.15766/mep_2374-8265.11308 (PMC10151448; doi:10.15766/mep_2374-8265.11308)
Supplement: Supplementary file 1 — Workshop Syllabus.docxExercise.docxWorkshop Introduction.pptxWorkshop Implementation Guide.docxPostsession Survey.docx [file mep_2374-8265.11308-s001.zip › D. Workshop Implementation Guide.docx]

**Appendix D: Values Clarification Workshop Implementation Guide**

This workshop is designed for first and second year medical students. It consists of a large group session, which any number of students can attend, followed by a small group session, during which students work in groups made up of 8-10 students and one or more facilitators. In order to run the workshop, organizers need to fill the following roles:

- A speaker to introduce the topic and goals of the workshop
  - See Appendix C for slides and notes for this brief presentation. In our case, one of the workshop designers gave the introduction.
- Two or more physician panelists to share what it was like to experience and manage a conflict between their personal values and their professional responsibilities.
  - The recruitment and preparation of the panelists is discussed in detail below.
- A moderator to interview the panelists, solicit questions from the audience, and ensure that the panel runs smoothly
  - In our case, one of the workshop designers performed this function.
- Small group facilitators
  - We recruited physicians, nurses, psychologists, social workers, bioethicists, and fourth-year medical students to serve as facilitators. We had sufficiently many facilitators that we were able to assign two to each group. As a result, many groups benefited from having facilitators from differing professions. However, we do not consider it a requirement that groups have multiple facilitators. Guidance for facilitators is included below.

**Panelists.** When recruiting panelists, we identified physicians who not only had experienced a profound conflict between their personal values and the expectations that their work placed on them, but who also recognized the benefit of sharing with students the process they undertook to address the conflict and the associated turmoil. In other words, we stressed to any potential panelist that we were not asking for an ethical analysis of a moral challenge they had faced but rather that we were interested in an account of their personal struggle and the choices they made that ultimately led to resolution. We found panelists by approaching physicians whom we knew had relevant experiences and who we believed would embrace the goals of this session (as they did). Certainly, recruitment through a less personal avenue, such as by sending out an email to those physicians already participating in medical education, could be effective. However, before deciding on a panelist, it is essential to have a conversation about the objectives of the workshop with that person. Given how far in advance physicians’ clinical schedules are set, and given the need to have at least one preparatory session as described below, we recommend recruiting panelists at least three months before the session.

Once we had chosen our panelists, we met with each of them to listen to their story and to focus their attention on the following prompts that would be posed to them during the workshop:

- Please describe a time when you experienced significant moral discomfort because your professional responsibilities and your deeply-held values were not aligned.
- Please describe the specific steps you took to resolve the tension you experienced. In particular, did you engage in a process of examining and clarifying your own values? If so, what did this introspective process look like and was it useful?
- How did you ultimately handle the conflict you felt? Did your discomfort dissipate as a result of the steps you took?
- Please offer any guidance you might have for our students, who are likely to experience this kind of discomfort at some point in the future.

We were especially careful in this preparatory meeting to make sure that our panelists understood the need to describe the reflection that they engaged in to clarify what was important to them and to share an identifiable resolution to the conflict they experienced. We recommend having this meeting 2-4 weeks before the session, as this gives the panelists some time to process the feedback, make adjustments, and return with questions if they have any. In our case, we did not meet with both panelists at the same time. However, doing so could result in panelists making changes to create a more cohesive, integrated presentation.

**Small group facilitators.**

*Pre-workshop*: We recommended that facilitators complete the assigned pre-work before the session, so that they would be familiar with what the students had been asked to do and would be able to comment on their own experience with the values clarification worksheet.

*Workshop, large group session*: Facilitators were required to attend the large group portion of the workshop.

*Workshop, small group session*:

5 minutes

Safe space guidelines: Facilitators had a special responsibility to ensure that the group was a safe environment for students. To this end, facilitators were reminded

- to be attentive to what students might be experiencing
- to present and uphold expectations of respectful discourse
- to encourage participation while ensuring that no individual felt pressure to share
- to intervene and redirect the conversation if the validity of someone's beliefs was being challenged

10 minutes

Check in: We suggested to facilitators that, if students were slow to participate, they get the ball rolling by going first. Each group member was expected to speak for about one minute. Everyone in the group was required to check in.

40 minutes

Groups were asked to choose two or three topics to discuss. As noted on the syllabus (Appendix A), the small group discussion was intended to give members of the group a chance to share their perspectives and concerns about healthcare topics that they may have found ethically challenging, to learn about the points of view and experiences of others in the group, to further clarify the nature of any conflict with professional expectations they had experienced or were envisioning, and to consider what steps they might take to manage a professional situation that pushed up against their personal values.

We believe strongly that for the small groups to be effective, students must be actively sharing their views and experiences. In support of this goal, we strongly urged facilitators to avoid dominating the conversation and instead asked them to be engaged participants whose primary role was to contribute their stories and perspectives to the conversation. Though we downplayed any special role for the facilitators, we did ask that they

- keep the conversation on track and moving, by offering comments and questions designed to provide direction and depth to the discussion
- manage time so that at least two topics could be addressed and checking out not be rushed
- be attentive to safety, as described above.

10 minutes

Check out with take-away points
