# Supplementary material for: Values Clarification as a Reflective Practice for Preclerkship Medical Students
Source: MedEdPORTAL. 2023 May 2;19:11308. doi: 10.15766/mep_2374-8265.11308 (PMC10151448; doi:10.15766/mep_2374-8265.11308)
Supplement: Supplementary file 1 — Workshop Syllabus.docxExercise.docxWorkshop Introduction.pptxWorkshop Implementation Guide.docxPostsession Survey.docx [file mep_2374-8265.11308-s001.zip › E. Postsession Survey.docx]

**Appendix E.** Values Clarification Workshop: Postsession Survey

| Survey Questions | Answer Choices |
| --- | --- |
| 1. This session helped me define a physician's professional responsibility with regard to one or more of these healthcare scenarios. | 1. Strongly Disagree 2. Disagree 3. Neutral 4. Agree 5. Strongly Agree |
| 2. The pre-work for this session was effective for this session. | 1. Strongly Disagree 2. Disagree 3. Neutral 4. Agree 5. Strongly Agree |
| 3. The large group session set the stage for the small group discussions. | 1. Strongly Disagree 2. Disagree 3. Neutral 4. Agree 5. Strongly Agree |
| 4. This session helped me understand and appreciate how, as a physician, I may experience conflict between my personal values and professional obligations. | 1. Strongly Disagree 2. Disagree 3. Neutral 4. Agree 5. Strongly Agree |
| 5. This session will help me better navigate situations in which my professional responsibility does not align with my personal beliefs. | 1. Strongly Disagree 2. Disagree 3. Neutral 4. Agree 5. Strongly Agree |
| 6. This session helped me recognize some of the ways in which my colleagues may have values and perspectives different than my own. | 1. Strongly Disagree 2. Disagree 3. Neutral 4. Agree 5. Strongly Agree |
| 7. This session was relevant to my future career as a physician. | 1. Strongly Disagree 2. Disagree 3. Neutral 4. Agree 5. Strongly Agree |
| 8. This session was an effective way to learn about the healthcare scenarios presented. | 1. Strongly Disagree 2. Disagree 3. Neutral 4. Agree 5. Strongly Agree |
| 9. When there is such a conflict, how could you use the approach from today's session to help you reconcile your personal beliefs and professional duties? | Short Answer |
| 10. What was the most meaningful component of this session? | Short Answer |
| 11. What is one way that this session could be improved? | Short Answer |
| 12. Any other comments you would like to provide? | Short Answer |
