# Supplementary material for: Values Clarification as a Reflective Practice for Preclerkship Medical Students
Source: MedEdPORTAL. 2023 May 2;19:11308. doi: 10.15766/mep_2374-8265.11308 (PMC10151448; doi:10.15766/mep_2374-8265.11308)
Supplement: Supplementary file 1 — Workshop Syllabus.docxExercise.docxWorkshop Introduction.pptxWorkshop Implementation Guide.docxPostsession Survey.docx [file mep_2374-8265.11308-s001.zip › Educational Summary Report_11308.pdf]

MedEdPORTAL® | The AAMC Journal of Teaching and Learning Resources

Original Publication

Open Access

Values Clarification as a Reflective Practice for Preclerkship Medical Students

Lloyd Chen*, Adrija Chaturvedi, Madeline McKenna, Mitchell Thom, MSc, Garrett Weskamp, Corinne Bazella, MD, Oliver Schirokauer, PhD, MD

*Corresponding author: lloyd.t.chen@gmail.com

Abstract

Introduction: Values clarification is a structured, reflective process individuals engage in to better understand their own beliefs and priorities. We designed a workshop on values clarification to help preclerkship medical students anticipate and manage potential conflicts between their personal values and professional expectations. **Methods:** We assigned participating students a values clarification exercise as prework. The 2-hour workshop included introductory remarks, a presentation by two physicians on personal ethical challenges they had faced, and faculty-facilitated small groups. In the small groups, students discussed moral discomfort in the context of various health care scenarios. Students were invited to complete an optional postworkshop survey with Likert-scale and short-answer questions. We analyzed the qualitative data and formulated 10 emerging themes. **Results:** Thirty-eight of 180 participating students (21%) returned the survey. Of these, 30 (79%) agreed the workshop helped them appreciate that their values might come into conflict with professional obligations, 26 (68%) agreed they would be able to apply what they learned to future scenarios, and 30 (79%) agreed the workshop helped them understand their colleagues' values. The most prominent themes identified were that students found the physician panel especially meaningful and that the workshop helped students examine their own values and prepared them to better understand their future patients' values. **Discussion:** Our workshop is unique in that it does not focus on a single area in health care but addresses moral discomfort broadly. To the best of our knowledge, it is the first values clarification curricular initiative developed for preclerkship medical students.

Keywords
Values Clarification, Personal Values, Clinical/Procedural Skills Training, Ethics/Bioethics, Professionalism, Reflection/Narrative Medicine

Educational Objectives

By the end of this workshop, learners will be able to:

1. Describe the origin and nature of their beliefs about two or more of the following topics: pregnancy termination, male circumcision, continuation/withdrawal of life-sustaining treatment, hormone therapy for transgender youth, physician aid in dying, and involuntary hospitalization.
2. Outline general steps to take when navigating a situation in which their professional responsibilities are in conflict with their personal moral beliefs and explain specifically how values clarification fits into this approach.
3. Describe the ways in which their beliefs align with or differ from those held by members of their discussion group regarding one or more of the six health care topics listed above.

Citation:
Chen L, Chaturvedi A, McKenna M, et al. Values clarification as a reflective practice for preclerkship medical students. *MedEdPORTAL*. 2023;19:11308. https://doi.org/10.15766/mep_2374-8265.11308

Introduction

We refer to the collection of deeply held moral beliefs and priorities that a person holds as their values. These values represent what is important to them in life and inform them of what the right thing to do is in any given situation. Values clarification is a reflective process that an individual undertakes to better understand the nature of their own values and how they have been shaped over time by key external influences, such as family, social groups, spiritual teachings, and personal experiences.[1] Values clarification exercises have been used in the medical field to prepare health care workers for clinical scenarios that are ethically complex and may challenge their personal beliefs.[1-3]

Copyright © 2023 Chen et al. This is an open-access publication distributed under the terms of the Creative Commons Attribution license.

We developed a workshop for first-year medical students that explores the role values play in the experience and practice of providing medical care. The workshop's primary goal is for students to appreciate the utility of values clarification as a reflective practice that can help them recognize, understand, and address the moral discomfort they may feel with the expectations placed on them professionally. A secondary aim is for students to learn about the backgrounds, experiences, and values that their colleagues bring to their work and that may be different from their own. Although the workshop focuses on moral challenges, it has not been designed to provide students with an ethical framework or ethics-based strategies for analyzing or resolving these tensions.

Professional identity formation is a core component of medical education. Students prepare to be doctors not only by learning the medical sciences and practicing clinical skills but also by developing a personal understanding of the nature and scope of medical practice and the obligations that come with being a physician.[4] This understanding is shaped by the clinical experiences that students have, including those scenarios in which they find themselves struggling with professional expectations that do not fully align with their personal values. Learning about and practicing values clarification during the preclerkship years can help prepare students to examine the complex emotions and concerns that arise in such situations and promote their ability to make decisions grounded in professionalism and self-knowledge. Our workshop, therefore, can be viewed both as preparation for the clinical years of medical school and as part of a broader professional development program.

Values clarification exercises have been designed to support clinical decision-making for a wide range of health care trainees and professionals, including residents,[2,5] clerkship medical students,[6-9] nurses,[3,10,11] social workers,[12] and health care providers in general.[1,13,14] Most of these resources focus on reproductive health,[1,2,5-9,13,14] including several that describe workshops targeted to clerkship medical students on their OB/GYN or women's health rotation.[6-9] Against this backdrop, our workshop contributes to values clarification medical education in two ways: First, because ethically complex situations occur throughout medicine, we do not restrict our attention to one specialty but instead promote the application of values clarification to many different health care scenarios; second, we offer a values clarification program developed for preclerkship medical students, thereby introducing the idea that this reflective approach can be used to lay a foundation for preclinical trainees to help them navigate the difficult situations that may lie ahead, perhaps even before they are done with medical school.

Methods

We piloted the values clarification workshop in the spring of 2021 for first-year medical students as part of a required course addressing a wide range of topics related to the patient-physician relationship and professional development. The syllabus (Appendix A) introduced students and facilitators to the workshop's requirements, learning objectives, and agenda. As preparation, we asked students to complete a values clarification exercise (Appendix B) and to read an article on conscientious objection.[15] The workshop itself was divided into two parts. During the first hour, students and facilitators heard a brief presentation about values clarification (Appendix C), which was followed by a much lengthier panel discussion in which two physicians each shared a story of a time when they felt conflict between their professional responsibilities and their values. During the second hour, students met in small groups to discuss their experience with the assigned values clarification exercise and their thoughts about managing professional expectations that they might find ethically uncomfortable.

We distributed the values clarification exercise to students via their online education portal and made clear to them that the worksheet was required and needed to be completed in order to have meaningful discussion during the workshop's small-group sessions. We adapted the exercise from a more extensive values clarification guide that had been created to explore values related to family planning.[1] Our version was intended to be completed in approximately an hour and consisted of a series of open-ended questions concerning student values, formative experiences, and perceptions of professional obligations. In particular, the worksheet asked students to reflect on the social and spiritual influences that had helped shape their value system and to consider the impact of any experiences they had encountered with six ethically complex medical topics (pregnancy termination, continuation/withdrawal of life-sustaining treatment, hormone therapy for transgender youth, physician aid in dying, involuntary hospitalization, and male circumcision). The worksheet ended by having students identify health topics they were uncomfortable with (not necessarily chosen from the list given earlier) and begin to formulate a process to address this discomfort. Space was provided throughout the worksheet for students to record their thoughts in writing. However, students were not asked to submit their responses to any of the questions in the exercise.

Copyright © 2023 Chen et al. This is an open-access publication distributed under the terms of the Creative Commons Attribution license.

This workshop took place during the COVID-19 pandemic, at a point when the medical school was beginning to return to in-person learning. The introductory remarks and physician panel were streamed for all students via teleconference. For a third of the students, the interactive, small-group portion of the workshop took place in person. The rest of the students participated in virtual small groups via teleconference.

The panel that addressed the full class consisted of two physicians who each spoke about a situation in which they had experienced profound moral discomfort regarding what was expected of them professionally. We did not expect the panelists to present a "correct" approach to such a crisis of conscience, but we did believe that for the integrity of the workshop, it was essential they describe a process that included a reflective component focused on gaining a deeper understanding of the origin and nature of their relevant values. Moreover, it was important that the process they adopted resulted in some change in their experience of the ethical challenge under consideration. During the workshop, the moderator elicited the first panelist's story and then proceeded to do likewise with the second panelist. Thus, each panelist in turn had a chance to describe the conflict they had experienced, how they came to understand their values more fully as a result, and how the situation was resolved. We organized the panel discussion in this way because we, as well as our panelists, felt their narratives would be more impactful if they were not entwined but were each told as a whole. However, in future implementations of the workshop, having panelists take turns responding to the various prompts posed to them could also be effective by allowing students to focus on different components of this sort of experience. Of note, we made sure that the topics our panelists addressed were on the list given to the students as part of their preworkshop exercise. We concluded the presentation by asking the first panelist, and then the second, to provide advice to the students about what to do when feeling morally troubled by the expectations placed on them. For more details about the recruitment and preparation of the panelists, including a list of the prompts to which they responded, refer to Appendix D.

Following the panel presentation, students met in small groups; each group had eight students and two facilitators. These groups had met frequently over the previous 8 months, and as a result, group members already had working relationships with one another. Though the group facilitators did not receive any specialized training for this workshop, they did receive substantial guidance during an extensive orientation at the beginning of the course that the workshop was a part of and during a subsequent facilitator meeting. At both gatherings, we stressed that the role of the facilitator was not that of a traditional instructor and urged facilitators to conceive of the small group as a setting in which all group members, especially students, would have the opportunity to share and explore their ideas and experiences. Appendix D contains more information about our expectations for facilitators.

At the outset of the values clarification discussion, each group reviewed the guidelines from the syllabus (Appendix A) for the creation of a safe discussion space. Each group then chose two or three health care topics to discuss. Students were asked to contribute to the conversation by sharing their experience of completing the values clarification exercise and by articulating the ways in which that experience may have impacted their comfort with the topic under consideration. Students were also prompted to relate any concerns of an ethical nature they might have regarding the professional expectations associated with the topic and to explore together what steps to take to address such concerns. The purpose of the discussion was to give students a chance to share their views in a safe environment, to learn about other perspectives and experiences, to further clarify the nature of any conflict they were envisioning, and to begin to consider with their peers how they might resolve the situation. They were explicitly and emphatically instructed not to debate the ethical merits of any particular point of view.

After the workshop, students were asked to complete an optional online survey (Appendix E), which contained both quantitative and qualitative items. We designed survey items to yield information about the quality of the workshop, its impact on the students, and whether the workshop's learning objectives (Appendix A and Educational Objectives above) had been achieved. In particular, survey items 1, 5, and 9 addressed the fulfillment of Educational Objective 2, and item 6 provided direct information regarding Educational Objective 3. No item explicitly evaluated the extent to which Educational Objective 1 had been met, though items 4 and 6 did so indirectly. Two of the remaining items (2 and 3) evaluated the effectiveness of particular workshop design elements, one item (8) inquired about learning not included in the Educational Objectives, and one item (7) was intended to measure the overall meaningfulness of the workshop. Survey items 10-12 were open-ended invitations to share what worked best and what needed improvement.

We performed a thematic analysis on the answers to the qualitative survey items using a methodology designed to categorize responses to open-ended questions.[16,17] Two authors independently coded the data using descriptive open coding

Copyright © 2023 Chen et al. This is an open-access publication distributed under the terms of the Creative Commons Attribution license.

methods to identify emergent codes, followed by a round of pattern coding, which consolidated content into categories based on shared or overlapping content. A third author reviewed the coding to resolve discrepancies.

Results

A total of 180 first-year medical students participated in the workshop. The quantitative portion of our postworkshop survey consisted of eight statements that students were asked to share their level of agreement with using a 5-point Likert scale (1 = *strongly disagree,* 5 = *strongly agree*). Of the 38 students (21% of attendees) who submitted responses to this part of the questionnaire, 37 rated all the statements, and one rated all but one statement. All the statements presented a positive view of the workshop. For every statement on the survey, the percentage of respondents who agreed or strongly agreed with the statement was between 68% and 89%, and for half of the statements, this percentage was greater than 75% (Figure).

The qualitative portion of our survey consisted of four short-answer questions. Of the 36 students (20% of attendees) who provided responses to one or more of these questions, 36 (100%) responded to the first survey question, 36 (100%) responded to the second question, 23 (64%) responded to the third question, and 13 (36%) responded to the fourth question. Thus, we obtained a total of 108 responses. Through thematic analysis, we identified 131 codes that were then categorized into the 10 major emerging themes displayed in the Table. We omitted 16 of the student responses from the analysis because their content was not substantive or specific enough to yield meaningful codes.

Discussion

Professional identity formation is an important component of medical education and is understandably often focused on fostering in students an understanding of the expectations of the medical profession. Our values clarification workshop is a novel initiative alerting students to the real possibility that the professional expectations they will encounter may not always sit well with them and giving them a chance to explore how their cultural, social, and spiritual backgrounds, as well as their life experiences, will shape the challenges of conscience that may lie ahead. As such, the workshop is meant to serve two interconnected purposes. First, it represents a step towards ensuring that students' professional identity is grounded in the knowledge of their own values, and second, it promotes the idea that such self-knowledge is useful for navigating ethical decision-making. The qualitative and quantitative data we collected

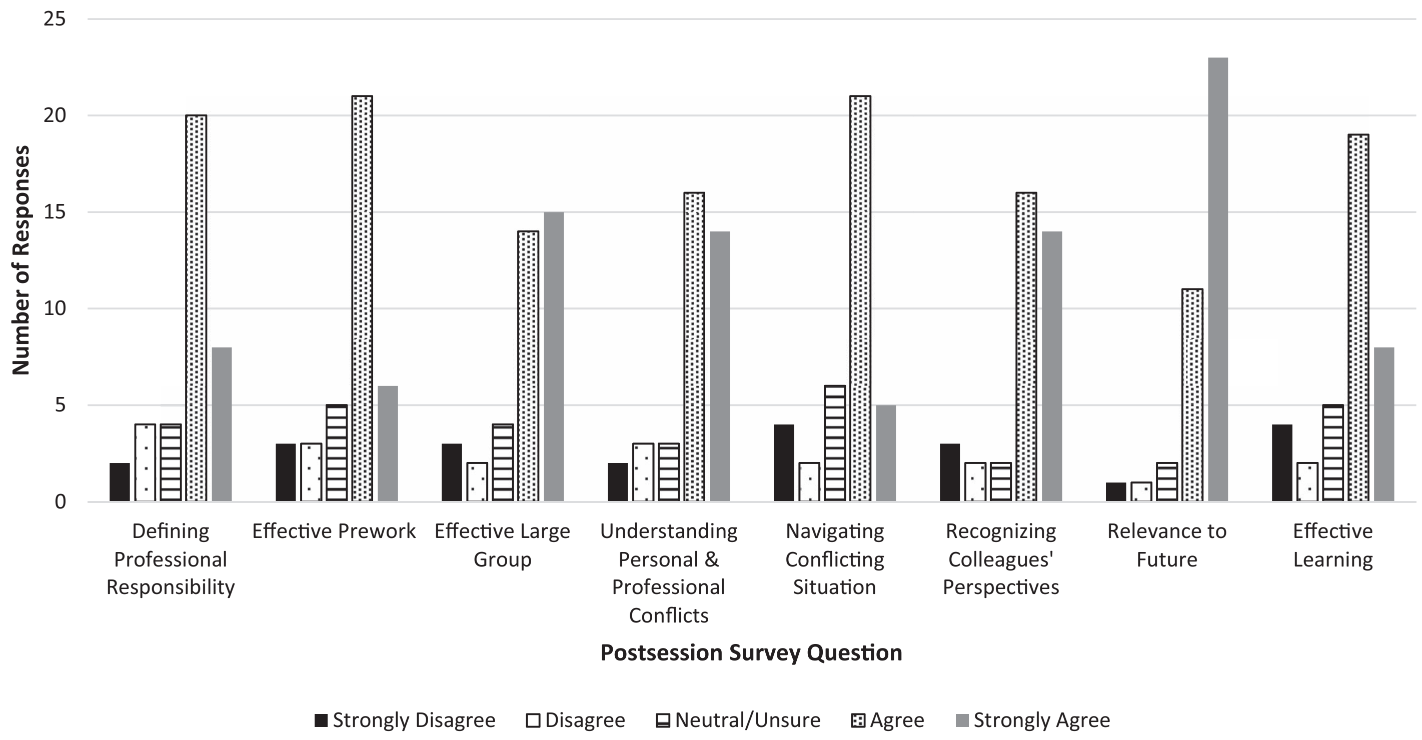

Figure. Bar chart of Likert-scale data from the postworkshop survey. For all questions, $n = 38$, except for question 6, where $n = 37$.

Copyright © 2023 Chen et al. This is an open-access publication distributed under the terms of the Creative Commons Attribution license.

Table. Themes Emerging From the Thematic Analysis of Postworkshop Survey Comments

Emerging Theme	No. (%) Comments[a]	No. (%) Responding Students[b]	Example Comments	Summary
1. Value of large-group session	21 (19)	19 (53)	"Hearing the physicians' challenging and intimate stories in the large group presentation was humbling and helped prepare us for a deeper discussion in our small groups."<br>"The speakers were really powerful. It really put things in context to not be talking in such a theoretical way."	Comments described the benefits of hearing physicians share specific and poignant examples of the role that values clarification plays in a career in medicine.
2. Growth as a future physician	18 (17)	17 (47)	"I think one of the big overarching themes I took away from the session was that improving the patient's situation comes first and foremost. While their choices may not be the ones I would make, helping them do so is paramount."	Comments described how the session shed light on the role of a physician in challenging patient scenarios and focused attention on understanding one's own values and those of one's patients.
3. Benefit of personal values clarification	18 (17)	16 (44)	"I can think more deeply about the root of my values so that I can come to a place where I made decisions not based on beliefs that I adopted from others, but on my own beliefs."	Comments described realizations about personal values arrived at during the clarification exercise and subsequent discussion, attesting to the utility of the framework for reflection and decision-making.
4. Value of perspective sharing	13 (12)	12 (33)	"I appreciated the chance to discuss values with my colleagues. Everyone was respectful and thoughtful despite the widely varying values. This is something that is vanishingly rare in today's value-laden discussions."	Comments described the benefits of learning about the varied experiences and diverse perspectives of other students.
5. Value of small-group session	13 (12)	11 (31)	"[The most meaningful component of this session was] getting to listen to responses and perspectives from my peers, while getting a chance to talk about my own values as well."	Comments described the benefits of the small-group session and, in particular, the important opportunity it provided to connect and share with one another.
6. Lack of clarity regarding professional obligations	9 (8)	9 (25)	"I don't recall learning a specific approach to reconcile my personal beliefs and professional duties. This would've been a good thing to go over in the lecture or discussion."	Comments expressed a desire to learn about clear-cut methods to sort through ethically difficult and personally challenging clinical situations.
7. Value of prework	8 (7)	7 (19)	"The paper provided for the session was a good argument that I can look back on if I am feeling conflicted."	Comments described the usefulness of the assigned exercise and reading as preparation for the class session and for navigating ethically challenging clinical situations.
8. Limited time	7 (6)	6 (17)	"The shortened small group time made it particularly hard to discuss all the issues that we were supposed to cover."	Comments expressed a desire for more time for discussion in both the large- and small-group settings.
9. Limited viewpoints expressed on controversial topics	6 (6)	4 (11)	"I think this activity was very one-sided and alienated many members of our class who do not subscribe to the popular perspectives on these issues. It certainly called for them to be either vulnerable in admitting that they hold unpopular views or to not dissemble about what they really think."	Comments expressed a concern that the learning environment was not conducive to having students openly share perspectives that they understood to be controversial and unpopular.
10. Concern for disadvantaged and minority groups	2 (2)	1 (3)	"I have the mindset that my morals should play no role on my duties. 'Morals' have been attributed to negligent care of BIPOC historically and is one of the primary mechanisms perpetuating the misogyny of pro-life movements. I think it would be beneficial to not beat around the bush and just say that in the pre-readings and large lecture."	Comments expressed a concern that discussions about morality divert attention away from considering the ways in which medicine has harmed disadvantaged and minority groups.

Abbreviation: BIPOC, Black, Indigenous, and people of color.
[a]Total number of comments = 108.
[b]Total number of responding students = 36.

suggest that the workshop benefited our students in the ways we intended.

At the heart of our workshop was a three-step progression. The first step was the assigned values clarification exercise that asked students to reflect on the development and nature of their beliefs and led them to consider the value-laden nature of medical practice and the impact that their values would have on their professional life. This introduction primed them to connect with the stories and views they heard during the workshop from the physician panelists and the members of their small groups and prepared them to share their own perspective. It also gave them a structured, reflective experience focused on themselves. Although the students will most likely forget the details of the

Copyright © 2023 Chen et al. This is an open-access publication distributed under the terms of the Creative Commons Attribution license.

exercise, they may internalize the idea that when confronted with difficult ethical decisions, careful self-inquiry can lead to a better understanding of where the challenge lies for them. In our postworkshop survey, 71% of the respondents agreed or strongly agreed that the prework, which also included an assigned reading, was effective preparation for the workshop. Additionally, in response to open-ended questions about the workshop, nearly one-fifth of respondents provided comments attesting to the value of the prework, and almost half submitted comments describing the benefits of values clarification. Though none of this data specifically addresses the impact of the assigned values clarification worksheet alone, these results suggest that for many, if not most, of the respondents, undertaking this exercise was a useful activity.

The second step was the physician panel, which served as the emotional engine of the workshop. In addition to sharing how one might handle a situation that elicits enormous moral discomfort, the presenters modeled for students what it was like to share one's struggles openly with others. As a result, students saw firsthand how worthwhile this sharing could be both for the person telling the story and for the listener. More than three-quarters (76%) of survey respondents agreed or strongly agreed that the large-group session set the stage for the small-group discussions that followed, and we received more comments attesting to the powerful impact of hearing physicians share their experiences than we did about any other identified theme.

The third and final step of the workshop was the time allotted to discussion. In their small groups, students had the opportunity to talk about their insights into their own values and their concerns about potentially difficult conflicts lying ahead. This part of the workshop solicited active participation and, we hope, deepened the individual's engagement with the topic and helped them feel they will be supported within the profession as they encounter morally uncomfortable situations. We received numerous comments from students stating that the chance to share their thoughts with the group was meaningful. Moreover, 79% of respondents agreed or strongly agreed that the workshop helped them recognize some of the ways in which their colleagues might have values and perspectives different than their own. Having them appreciate the breadth of beliefs held by others, even within their own cohort, was another of our articulated goals.

Implementing the three components of the values clarification workshop described above came with various challenges. For instance, for the values clarification worksheet to be successful, students needed to put time and energy into completing it. However, ensuring that this happened was difficult, especially since asking students to submit answers to the exercise's questions, which were of a personal and private nature, would have been counterproductive. Including the physician panel required finding one or more clinicians willing to speak about a time when they had to sort out a personal-professional values conflict. We believe that many physicians have such stories to share but also recognize that recruiting suitable participants may require some effort. Regarding the small-group discussion, it was important that there was sufficient trust among the members of the small groups so that students were comfortable expressing views that might not be held by others. In our case, the longitudinal nature of the course that this workshop was a part of meant that these groups were well established. Implementing the workshop as a stand-alone event would require paying special attention to articulating and establishing suitable norms at the outset of the small-group work. Finally, we note that the workshop required a substantial time commitment. We had 2 hours, which was adequate but by no means excessive.

There are a number of ways in which our workshop could be improved. Despite all our attempts to ensure a safe space for students, we received explicit feedback that some learners were reluctant to talk about their experiences and perspective and felt that their views were delegitimized. A priority going forward is to review the workshop with an eye to doing even more to create a safe environment for participants. As part of this effort, one change we plan to make is to communicate more extensively with faculty facilitators in advance about the need to redirect the conversation if the validity of a student's values is being challenged.

A second concern for some students was the absence of clear-cut guidelines about how to reconcile conflicts between personal beliefs and professional duties. Here, we encounter the difficulty that the workshop is designed to increase students' awareness about such conflicts and to offer a way to engage with them that is admittedly nonspecific and nondirective. In future years, we will pay greater attention to communicating the goals of the workshop and will stress the general nature of its lessons. Moreover, though we have included in the introductory remarks some comments on clarification of professional expectations as the companion to clarification of personal values, we will explain more clearly that values clarification is only one component of the work involved in managing the sort of ethical conflicts under consideration. Lastly, we will make sure that our panelists describe explicitly the steps they took to resolve the ethical tension that they experienced, so that students see what a path out of a moral quandary might look like.

Copyright © 2023 Chen et al. This is an open-access publication distributed under the terms of the Creative Commons Attribution license.

A third area that deserves comment is student assessment. In our case, this workshop was part of a longitudinal course in which students received periodic oral and written feedback from faculty about their performance in the course and about the written reflections they were asked to submit after every eight to 10 sessions. Thus, there were opportunities for faculty to engage with students about the values clarification workshop and the learning that resulted. However, these avenues for assessment were not specific to the workshop. Instead, our approach was largely to expect that students would familiarize themselves with the learning objectives presented to them on the syllabus and subsequently attend to and assess their own learning. Educators who are implementing this program, especially as a stand-alone workshop, may want students to complete a postworkshop writing assignment in which they are asked to comment on the impact of listening to the experiences of the panelists and their peers as well as to describe the values clarification process and its potential role in their professional lives.

Our project has some important limitations. To begin with, the low response rate to the survey (21% for the Likert-scale questions, 20% for the short-answer part) raises the critical question of whether the responses we received reflect the views of the class more generally. In addition, we did not obtain any demographic data about the students who responded and therefore cannot draw any conclusions about whether they are demographically representative of the class. Another gap in our analysis is our lack of information about the extent to which students completed the assigned values clarification worksheet. Although attendance at the workshop was mandatory, students were not asked to submit any preparatory work and were not asked on the postworkshop survey about whether they had prepared for the workshop. An additional concern is that our survey was not designed to include, for every Educational Objective, an item directly inquiring about that objective. We plan to address these shortcomings in future workshops by finding ways to motivate students to respond to the survey and by modifying the survey to include demographic questions, a question about completion of the prework, and questions that explicitly align with each of the workshop's Educational Objectives. Finally, when we revise the survey, we will also change the wording of questions 1 and 8 in order to clarify that we are referring to the health care scenarios listed at the beginning of the values clarification worksheet and on the syllabus.

In conclusion, we offer a curricular initiative for preclerkship medical students that aims to prepare them to navigate times when the professional expectations placed on them do not align with their personal values. We were intent on including this workshop in the preclerkship curriculum because we had found that already in their clerkships, some students were struggling with these difficult conflicts. That being said, we believe that this workshop, built around three core activities, can be useful for health care practitioners at any stage of their careers and across different professions.

Appendices

A. Workshop Syllabus.docx

B. Exercise.docx

C. Workshop Introduction.pptx

D. Workshop Implementation Guide.docx

E. Postsession Survey.docx

All appendices are peer reviewed as integral parts of the Original Publication.

Lloyd Chen: Fourth-Year Medical Student, Case Western Reserve University School of Medicine; ORCID: https://orcid.org/0000-0002-3617-3785

Adrija Chaturvedi: Fourth-Year Medical Student, Case Western Reserve University School of Medicine

Madeline McKenna: Fourth-Year Medical Student, Case Western Reserve University School of Medicine

Mitchell Thom, MSc: Fourth-Year Medical Student, Case Western Reserve University School of Medicine

Garrett Weskamp: Fourth-Year Medical Student, Case Western Reserve University School of Medicine

Corinne Bazella, MD: Associate Professor, Department of Reproductive Biology, Case Western Reserve University School of Medicine

Oliver Schirokauer, PhD, MD: Assistant Professor, Department of Bioethics, Case Western Reserve University School of Medicine

Acknowledgments
Authors Adrija Chaturvedi, Madeline McKenna, Mitchell Thom, and Garrett Weskamp contributed equally.

Disclosures
None to report.

Funding/Support
None to report.

Prior Presentations
Chaturvedi A, Chen L, McKenna M, et al. Values clarification as a reflective practice for first-year medical students. Poster presented

Copyright © 2023 Chen et al. This is an open-access publication distributed under the terms of the Creative Commons Attribution license.

virtually at: Center for the Advancement of Medical Learning Annual Education Retreat; April 29, 2021.

Ethical Approval

The Case Western Reserve University Institutional Review Board deemed further review of this project not necessary.

References

1. *The Abortion Option: A Values Clarification Guide for Health Care Professionals*. National Abortion Federation; 2005.
2. Orr DP, Vickery ML. A values clarification workshop experience for residents. *J Adolesc Health Care*. 1983;3(4):256-263. https://doi.org/10.1016/S0197-0070(83)80248-4
3. Uustal DB. Values clarification in nursing: application to practice. *Am J Nurs*. 1978;78(12):2058-2063. https://doi.org/10.1097/00000446-197812000-00029
4. Pawlina W. Not "How should I learn?" or "How should I act?" but, "Who shall I become?": a précis on the roots of early professional identity formation in the anatomy course. *Anat Sci Educ*. 2019;12(5):465-467. https://doi.org/10.1002/ase.1914
5. Prine L, Lesnewski R, Bregman R. Integrating medical abortion into a residency practice. *Fam Med*. 2003;35(7):469-471.
6. Lupi C, Broome M, Runyan A, Schreiber N. Conscientious refusal: a workshop to promote reflective and active learning of ethics, communication skills and professionalism. *MedEdPORTAL*. 2012;8:9077. https://doi.org/10.15766/mep_2374-8265.9077
7. Lupi CS, Ward-Peterson M, Castro C. Non-directive pregnancy options counseling: online instructional module, objective structured clinical exam, and rater and standardized patient training materials. *MedEdPORTAL*. 2017;13:10566. https://doi.org/10.15766/mep_2374-8265.10566
8. Chaudhari S, Zuo S, Robinson J, Patel S. Medical students encounter values conflicts frequently in clerkships: a need for values clarification education [5C]. *Obstet Gynecol*. 2019;133(suppl):32S. https://doi.org/10.1097/01.AOG.0000559426.99880.ba
9. Steinauer J, Sufrin C, Hawkins M, Koenemann K, Preskill F, Dehlendorf C. Caring for challenging patients workshop. *MedEdPORTAL*. 2014;10:9701. https://doi.org/10.15766/mep_2374-8265.9701
10. Stephany K. *The Ethics of Care: A Moral Compass for Canadian Nursing Practice*. Bentham Books; 2012:58-70.
11. *Exploring Values & Beliefs to Create a Shared Purpose*. Foundation of Nursing Studies; 2015. Accessed February 10, 2023. https://www.fons.org/resources/documents/Creating-Caring-Cultures/CreativeApproachesVC.pdf
12. Bent-Goodley TB. Teaching social work students to resolve ethical dilemmas in domestic violence. *J Teach Soc Work*. 2007;27(1-2):73-88. https://doi.org/10.1300/J067v27n01_05
13. Kidd R, Clay S, Chiiya C. *Understanding and Challenging HIV Stigma: Toolkit for Action*. Rev ed. International HIV/AIDS Alliance/Academy for Educational Development/International Center for Research on Women; 2007. Accessed February 10, 2023. https://www.icrw.org/publications/understanding-and-challenging-hiv-stigma-toolkit-for-action/
14. Turner KL, Pearson E, George A, Andersen KL. Values clarification workshops to improve abortion knowledge, attitudes and intentions: a pre-post assessment in 12 countries. *Reprod Health*. 2018;15:40. https://doi.org/10.1186/s12978-018-0480-0
15. Stahl RY, Emanuel EJ. Physicians, not conscripts—conscientious objection in health care. *N Engl J Med*. 2017;376(14):1380-1385. https://doi.org/10.1056/NEJMsb1612472
16. Hsieh HF, Shannon SE. Three approaches to qualitative content analysis. *Qual Health Res*. 2005;15(9):1277-1288. https://doi.org/10.1177/1049732305276687
17. Elo S, Kyngäs H. The qualitative content analysis process. *J Adv Nurs*. 2008;62(1):107-115. https://doi.org/10.1111/j.1365-2648.2007.04569.x

Received: June 27, 2022
Accepted: December 29, 2022
Published: May 2, 2023

Copyright © 2023 Chen et al. This is an open-access publication distributed under the terms of the Creative Commons Attribution license.
